# Supplementary material for: Work-Related Exposures and Sickness Absence Trajectories: A Nationally Representative Follow-up Study among Finnish Working-Aged People
Source: Int J Environ Res Public Health. 2019 Jun 13;16(12):2099. doi: 10.3390/ijerph16122099 (PMC6616391; doi:10.3390/ijerph16122099)
Supplement: Supplementary file 1 [file ijerph-16-02099-s001.pdf]

Table S1. Fit statistics for sickness absence trajectories with a quadratic shape. Selected model in bold.

No. of Trajectories	AIC [a]	BIC [b] *N* = 3814	SSABIC [c] *N* = 24596	Latent Class Proportions (%)	Posterior Probability
2	−10517.7	−10549.1	−10558.3	75/25	0.94/0.85
**3**	**−10480.1**	**−10517.6**	**−10528.8**	**53/35/12**	**0.69/0.85/0.84**
4	−10450.2	−10506.1	−10523.2	20/56/10/14	0.78/0.65/0.64/0.83

[a] AIC = Akaike Information Criterion, [b] BIC = Bayesian Information Criterion in subject level, [c] SSABIC = Sample Size Adjusted Bayesian Information criterion.

Table S2. Fit statistics for five best three-trajectory models. Selected model in bold.

Trajectory Shapes [a]	AIC [b]	BIC [c] *N* = 3814	SSABIC [d] *N* = 24596	Latent Class Proportions (%)	Posterior Probability
001	−10505.1	−10533.2	−10541.6	56/34/10	0.79/0.76/0.77
**010**	**−10486.0**	**−10514.1**	**−10522.5**	**54/34/12**	**0.71/0.83/0.83**
110	−10486.9	−10518.2	−10527.5	56/33/11	0.73/0.82/0.83
111	−10485.7	−10520.0	−10530.3	56/33/11	0.72/0.83/0.84
112	−10483.8	−10521.3	−10532.5	55/33/12	0.70/0.84/0.83

[a] Trajectory shapes: 0 = intercept, 1 = linear, [b] AIC = Akaike Information Criterion, [c] BIC = Bayesian Information Criterion in subject level, [d] SSABIC = Sample Size Adjusted Bayesian Information Criterion.

Table S3. Risk and protective factors in association with three sickness absence trajectories. (Musculoskeletal disorders are adjusted for).

Summary exposure	All	Low	Slowly increasing	High	Full model * Trajectory Slowly increasing vs. Low		Full model * Trajectory High vs. Low	
	N	*N*	*N*	*N*	OR [α]	95% CI [β]	OR [α]	95% CI [β]
Factors that decrease the risk of sickness absence								
Prolonged sitting or keyboard use								
Neither	2207	1130	784	293	1		1	
Either	763	449	241	73	0.78	0.65-0.94	0.65	0.49-0.87
Both	844	504	262	78	0.70	0.58-0.84	0.53	0.40-0.71
Number of factors that increase the risk of sickness absence (nine work factors[€]*)*								
0	1258	770	391	97	1		1	
1	992	541	346	105	1.28	1.06-1.54	1.42	1.05-1.92
2−3	769	405	260	104	1.28	1.05-1.57	1.78	1.30-2.44
≥4	795	367	290	138	1.68	1.37-2.07	3.04	2.24-4.12

[α] Odds ratio, [β] 95 % Confidence interval, [*]ORs adjusted for age (continuous), gender, basic education, marital status, BMI, smoking, leisure time physical activity, alcohol dependence, job strain, social support at work, sleep problems, physical disorders excluding musculoskeletal causes, and mental disorders. [€] Prolonged standing, repetitive arm movement, arms above shoulder level, bent postures, squatting or kneeling, using a vibrating tool, high hand grip force, frequent handling of loads at least 5 kg, handling of loads at least 20 kg.

Table S4. Risk and protective factors combined in association with three sickness absence trajectories. (Musculoskeletal disorders not adjusted for).

Combined Exposure	Low	Slowly Increasing	High	Full Model * Trajectory Slowly Increasing vs. Low		Full Model * Trajectory High vs. Low	
	N	*N*	*N*	OR [α]	95% CI [β]	OR [α]	95% CI [β]

Prolonged sitting and keyboard use or physically demanding work factors							
Neither	290	169	48	1		1	
Prolonged sitting or keyboard use only	480	222	49	0.78	0.61–1.01	0.58	0.37–0.89
Physically demanding work factors[€] only	840	615	245	1.30	1.04–1.63	1.61	1.14–2.28
Both	473	281	102	1.02	0.80–1.31	1.11	0.76–1.63

[α] Odds ratio, [β] 95 % Confidence interval, * ORs adjusted for age (continuous), gender, basic education, marital status, BMI, smoking, leisure time physical activity, alcohol dependence, job strain, social support at work, sleep problems, physical disorders excluding musculoskeletal causes, and mental disorders. [€] Prolonged standing, repetitive arm movement, arms above shoulder level, bent postures, squatting or kneeling, using a vibrating tool, high hand grip force, frequent handling of loads at least 5 kg, handling of loads at least 20 kg.

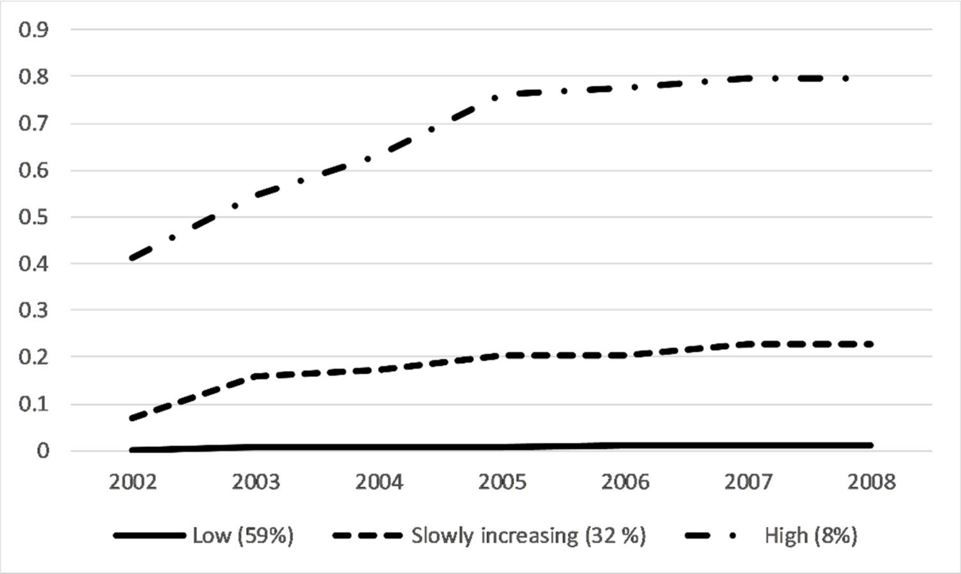

Figure S1. Three sickness absence trajectories among 30–44-year old women and men in the follow-up from 2002 to 2008; 1 = low 2 = increasing, and 3 = high (x-axis: the follow-up from 2002 through 2008, y-axis = annual number of sickness absence periods).

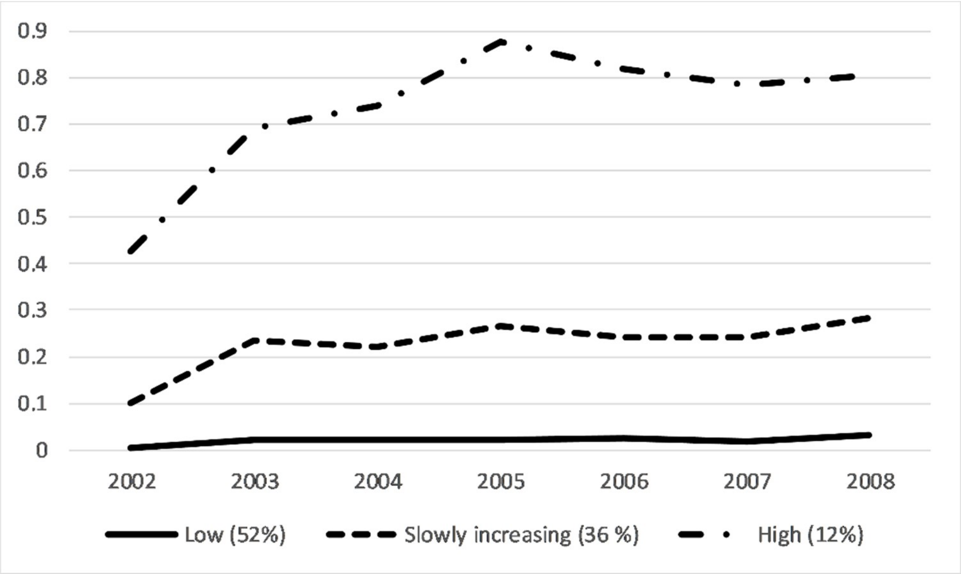

Figure S2. Three sickness absence trajectories among 45–59-year old women and men in the follow-up from 2002 to 2008; 1 = low 2 = increasing, and 3 = high (x-axis: the follow-up from 2002 through 2008, y-axis = annual number of sickness absence periods).
